# Diagnostic value of CRP, PCT, NC, and NLR in peripheral blood for bacterial infections in non-small cell lung cancer patients after chemotherapy

**DOI:** 10.5937/jomb0-57772

**Published:** 2025-10-28

**Authors:** Bin Wang, Yanbin Wei

**Affiliations:** 1 The 4th Centre Hospital of Nankai University, Department of Thoracic Surgery, Tianjin, China; 2 Lishui Second People's Hospital, Department of Pulmonary and Critical Care Medicine, Lishui, Zhejiang, China

**Keywords:** non-small cell lung cancer, bacterial infections, CRP, PCT, neutrophil count, NLR, nemikrocelularni karcinom pluća, bakterijske infekcije, CRP, PCT, broj neutrofila, NLR

## Abstract

**Background:**

This study aimed to evaluate and compare the diagnostic value of C-reactive protein (CRP), procalcitonin (PCT), neutrophil count (NC), and neutrophil-to-lymphocyte ratio (NLR) in peripheral blood for detecting bacterial infections in patients with non-small cell lung cancer (NSCLC) following chemotherapy.

**Methods:**

A total of 122 NSCLC patients treated at our hospital between October 2021 and October 2024 were enrolled. Of these, 72 patients with confirmed bacterial infections post-chemotherapy were assigned to the infection group, while 50 patients without infections were included in the non-infection group. General clinical data, overall survival, and levels of CRP, PCT, NC, and NLR were compared between groups.

**Results:**

Levels of CRP, PCT, NC, and NLR were significantly higher in the infection group compared to the non-infection group (P&lt;0.05). There was no significant difference in overall survival between the two groups (P=0.749). Receiver operating characteristic (ROC) curve analysis showed that all four biomarkers had statistically significant diagnostic value (P&lt;0.05), with PCT demonstrating the highest AUC (1.000), followed by NLR (0.981).

**Conclusions:**

PCT and NLR are valuable biomarkers for diagnosing bacterial infections in NSCLC patients after chemotherapy. Due to their complementary diagnostic strengths, PCT offers high specificity and NLR high sensitivity, and their combined use may enhance early detection and improve clinical decision-making.

## Abstract (sr)

**Uvod:**

Cilj ove studije je bio da se proceni i uporedi dijagnostička vrednost C-reaktivnog proteina (CRP), prokalcitonina (PCT), broja neutrofila (NC) i odnosa neutrofila i limfocita (NLR) u perifernoj krvi za otkrivanje bakterijskih infekcija kod pacijenata sa nemikrocelularnim karcinomom pluća (NSCLC) nakon hemioterapije.

**Metode:**

U studiju su bila uključena ukupno 122 pacijenta sa NSCLC lečena u našoj bolnici između oktobra 2021. i oktobra 2024. godine. Od toga je 72 pacijenata sa potvrđenim bakterijskim infekcijama nakon hemioterapije svrstano u grupu sa infekcijom, dok je 50 pacijenata bez infekcije uključeno u grupu bez infekcije. Upoređeni su opšti klinički podaci, ukupno preživljavanje i nivoi CRP , PCT, NC i NLR između grupa.

**Rezultati:**

Nivoi CRP , PCT, NC i NLR bili su značajno viši u grupi sa infekcijom u poređenju sa grupom bez infekcije (P&lt;0,05). Nije bilo značajne razlike u ukupnom preživljavanju između dve grupe (P=0,749). Analiza ROC krive je pokazala da su sva četiri biomarkera imala statistički značajnu dijagnostičku vrednost (P&lt;0,05), pri čemu je PCT imao najveću površinu ispod krive (AUC 1,000), a zatim NLR (0,981).

**Zaključak:**

PCT i NLR su značajni biomarkeri za dijagnostikovanje bakterijskih infekcija kod pacijenata sa NSCLC nakon hemioterapije. Zbog svojih komplementarnih dijagnostičkih svojstava - PCT pruža visoku specifičnost, a NLR visoku senzitivnost - njihova kombinovana upotreba može poboljšati rano otkrivanje infekcija i unaprediti kliničko odlučivanje.

## Introduction

Lung cancer (LC) remains the leading cause of cancer-related morbidity and mortality worldwide, with non-small cell lung cancer (NSCLC) accounting for approximately 85% of all LC cases [1] [2] [3] [4]. While early-stage NSCLC can often be managed effectively with surgical intervention, the overall 5-year survival rate remains below 20% [5] [6], primarily due to delayed diagnosis. More than 75% of patients are diagnosed at advanced stages when curative surgery is no longer an option. Platinum-based chemotherapy continues to be the cornerstone of first-line treatment for advanced NSCLC, and although some patients achieve remission, outcomes are frequently compromised by treatment-associated complications [7] [8].

One of the most significant complications is the high risk of nosocomial (hospital-acquired) bacterial infections during chemotherapy. These infections are particularly prevalent in advanced NSCLC patients due to their immunocompromised state, reduced organ function, and the myelosuppressive effects of cytotoxic drugs. Such infections not only increase hospitalisation time and healthcare costs but also hinder treatment continuity, reduce chemotherapy efficacy, and can even result in mortality [9].

Despite their clinical importance, bacterial infections during chemotherapy are notoriously difficult to detect early, as their symptoms often overlap with cancer-related systemic effects or chemotherapyinduced side effects [10] [11]. Traditional infection markers such as white blood cell count and C-reactive protein (CRP) are commonly used but may lack specificity in this context.

In recent years, procalcitonin (PCT) and the neutrophil-to-lymphocyte ratio (NLR) have emerged as more promising biomarkers for the early detection of bacterial infections. PCT, a precursor of the hormone calcitonin, is known to rise specifically in response to bacterial infections, distinguishing them from viral or inflammatory causes [12] [13]. Meanwhile, NLR – a ratio derived from routine blood counts – reflects the balance between neutrophil-driven inflammation and lymphocyte-mediated immune regulation. Elevated NLR has been shown to correlate with bacterial infections and has demonstrated superior diagnostic value over some traditional markers in oncology patients [14].

Therefore, this study aims to evaluate the diagnostic utility of CRP, PCT, neutrophil count, and NLR in identifying bacterial infections in patients with advanced NSCLC undergoing chemotherapy. By clarifying their clinical relevance, we hope to contribute to improved infection management strategies, better prognosis, and enhanced quality of life for this vulnerable patient population.

## Materials and methods

### General data

The 122 NSCLC patients under treatment in our hospital from October 2021 to October 2024 were selected as the research cohort; among them, 72 NSCLC patients with bacterial infections after chemotherapy were chosen as the infection group, and 50 NSCLC patients without bacterial infection after chemotherapy received selection as non-infection group. All research cohorts signed informed consent and voluntarily participated in this research. The research plan has received approval from the hospital’s medical ethics committee. Inclusion criteria: All NSCLC patients met diagnostic criteria for NSCLC in the Clinical Practice Guidelines for Non-Small Cell Lung Cancer (5th Edition, 2017) developed by the National Comprehensive Cancer Network (NCCN) in the United States [15]; clinical staging met staging criteria for NSCLC stage III-IV in the Clinical Standards for Tumor Staging (8th Edition, 2017) established by the American Joint Committee on Cancer (AJCC) and the International Union for Cancer Control (UICC) [16]; all patients underwent hospitalisation for at least one course of chemotherapy; complete clinical data; expected survival period was greater than 3 months. All patients in infection group met Nosocomial Infection Diagnosis Standards formulated by the National Health Commission; patients received confirmation as bacterial infection through positive bacterial culture results of clinical specimens (e.g., sputum, blood, urine) combined with imaging findings such as chest CT or X-ray showing localised infiltrates consistent with bacterial pneumonia; non-infection patients received exclusion from nosocomial infection through clinical examination and negative microbiological testing; control group received exclusion from malignancies and bacterial infections through clinical examination. Exclusion criteria: (1) Those with concomitant heart and brain stroke, primary liver and kidney dysfunction, haematological disorders and immunodeficiency; (2) those who had been diagnosed with the bacterial infection before admission; (3) those who had received antitumour therapy before enrollment; (4) those with tumour recurrence, distant metastasis, or concomitant extrapulmonary primary malignancies.

### Observation indicators

(1) General data. The age, gender composition, and comorbidities of all research subjects were compared by reviewing their admission medical records and physical examination registration forms. The pathological types and tumour staging of patients in infection and non-infection groups were compared by reviewing their admission medical records. Among them, diagnosis of diabetes, hypertension, coronary heart disease, and chronic obstructive pulmonary disease (COPD) was based on the Medical Diagnosis and Treatment Standards for diabetes (2019) [17], the International Hypertension Practice Guidelines (2020) [18], the Diagnosis and Treatment Guidelines for Stable Coronary Heart Disease (2012) [19], and the Global Strategy for Diagnosis, Management, and Prevention of Chronic Obstructive Pulmonary Disease (2017) [20]. The NSCLC pathological types and tumour staging were based on the Clinical Standards for Tumor Staging (8th Edition, 2017) [16].

(2) CRP and PCT levels. The 4 mL fasting peripheral venous blood specimens received collection from all subjects in duplicate, one of which received anticoagulation treatment. The blood collection time for the infection group was the day of confirmed bacterial infection, typically occurring 5 to 10 days after chemotherapy; the blood collection time for the non-infection group was standardised as day 7 post-chemotherapy, corresponding to the expected period of bone marrow suppression; blood collection time for the control group was the day of physical examination. Blood specimens without anticoagulation treatment were taken, let stand at room temperature for coagulation, and received centrifugation at a speed of 3000 r/min for 10 min to prepare serum specimens. The A5000 dry fluorescence immunoassay analyser was utilised to detect whole blood CRP and serum PCT levels through dry immunofluorescence quantification.

(3) Peripheral blood NC and NLR levels. The anticoagulant whole blood specimens were taken,and the Bayer ADVIA 2120 automatic blood analyser (Bayer Company, Germany) was utilised to test blood routine indicators. The principle of combining flow cytometry and laser counting was utilised for blood routine five classification detection and analysis.

(4) Overall survival. The overall survival in both groups was recorded.

### Statistical analysis

SPSS 27.0 software and GraphPad Prism 9.0 were utilised for statistical data analysis. Measurement data in line with normal distribution received representation with (x̄ ± s). Multiple-group comparisons were conducted through analysis of variance, with inter-group comparison through the SNK-q test. The counting data received representation with [n (%)], with inter-group comparison through the χ^2^ test. The diagnostic value of indicators was analysed via the receiver operating characteristic (ROC) curve, with the area under the curve (AUC) as the evaluation basis. The overall survival rate received was calculated using Kaplan-Meier analysis. P<0.05 indicated a statistically significant difference.

## Results

### There was no marked difference in both groups in terms of general data

The age, gender, underlying diseases, pathological types and clinical stages demonstrated no difference between infection and non-infection groups (P>0.05; Table 1).

**Table 1:** General data in two groups.

Groups		Non-infection group	Infection group	χ^2^/t	P
N		50	72		
Gender<br>[n (%)]	Male	40 (80.0)	60 (83.3)	0.222	0.638
	Female	10 (20.0)	12 (16.7)		
Age (years)		59.04±8.14	59.79±8.66	0.51	0.611
Underlying diseases<br>[n (%)]	Diabetes mellitus	8 (16.0)	10 (13.9)	0.832	0.842
	Hypertension	18 (36.0)	20 (27.8)		
	Coronary disease	6 (12.0)	11 (15.3)		
	COPD	5 (10.0)	8 (11.1)		
Pathological types<br>[n (%)]	Squamous cell carcinoma	32 (64.0)	45 (62.5)	0.029	0.866
	Adenocarcinoma	18 (36.0)	27 (37.5)		
Clinical stages<br>[n (%)]	III	28 (56.0)	44 (61.1)	0.319	0.572
	IV	22 (44.0)	28 (38.9)		

### Comparison of CRP, PCT, NC and NLR levels in peripheral blood between both groups

**Figure 1:**
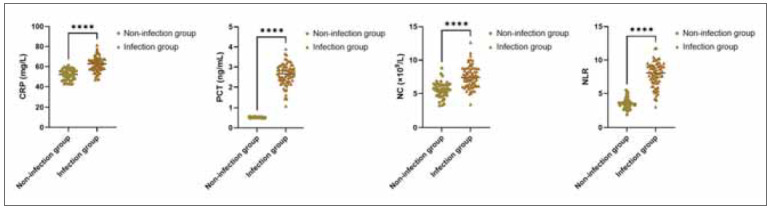
CRP, PCT, NC and NLR levels in three groups.<br>Note: Versus the non-infection group, ****P<0.0001.

The CRP, PCT, NC, and NLR levels in the infection group demonstrated an elevation in comparison to those in the non-infection group (P<0.05; Figure 1).

### Comparison of overall survival between both groups

**Figure 2:**
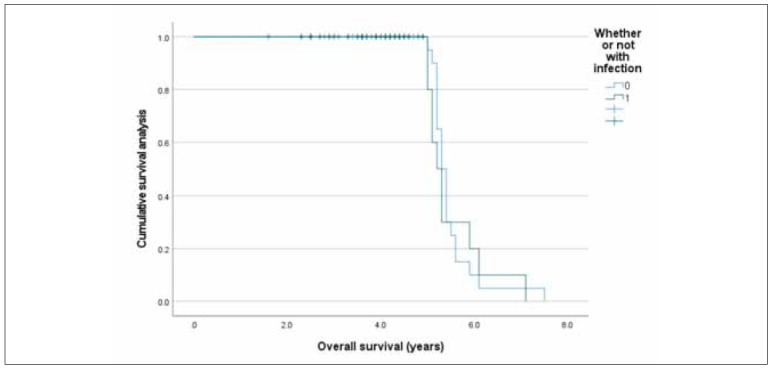
Overall survival in both groups through Kaplan Meier.

The overall survival demonstrated no difference in the infection group in comparison to that in the non-infection group (P=0.749; Figure 2).

### ROC curve of peripheral blood CRP, PCT, NC and NLR in diagnosing bacterial infections in NSCLC patients after chemotherapy

**Figure 3:**
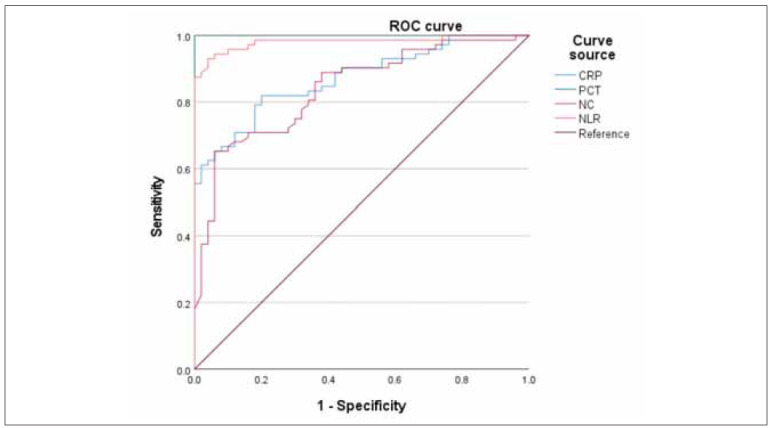
ROC curve of four indicators in diagnosing bacterial infections in NSCLC patients after chemotherapy.

Among four blood biomarkers, ROC curves of CRP, PCT, NC and NLR diagnosing bacterial infection in NSCLC patients after chemotherapy were represented in Figure 3.

### Comparison of the predictive value of CRP, PCT, NC and NLR levels in diagnosing bacterial infections in NSCLC patients after chemotherapy

The AUC values of CRP, PCT, NC and NLR for diagnosing bacterial infections in NSCLC patients after chemotherapy received comparison, illustrating statistical significance (P<0.05; Table 2). Among them, PCT had the highest AUC value of 1.000, and next was the AUC value of NLR (0.981; Table 2).

**Table 2:** Comparison of predictive value of CRP, PCT, NC and NLR levels in diagnosing bacterial infections in NSCLC patients after chemotherapy.

Groups	AUC	SE	P	95% CI	Cutoff value	Sensitivity	Specificity
CRP (mg/L)	0.871	0.06	<0.001	0.418–0.655	8.14	30.00%	96.00%
PCT (ng/mL)	1	0.023	<0.001	0.918–1.000	0.92	94.00%	98.00%
NC (×10^9^/L)	0.84	0.049	<0.001	0.642–0.835	6.35	66.00%	72.00%
NLR	0.981	0.031	<0.001	0.856–0.979	4.82	88.00%	88.00%
χ^2^	66.787	/	/	/	/	118.646	41.169
P	<0.001	/	/	/	/	<0.001	<0.001

## Discussion

Currently, therapy for advanced NSCLC in clinical practice is majorly based on internal medicine. Though new treatment technologies such as targeted therapy and immunotherapy have made remarkable advancements in recent years, the first-line therapy plan for advanced NSCLC still relies majorly on chemotherapy. It has been demonstrated that various elements are influencing the progression-free survival of NSCLC patients after chemotherapy, including not only the clinical stage but also changes in tumour markers; it also includes the number of chemotherapy courses completed and median progression-free survival of patients who can complete 4–6 courses depicts elevation relative to those who only complete 1–3 courses [21]. Thus, mitigating chemotherapy side effects and complications, elevating patient tolerance to chemotherapy, and ensuring sufficient therapy cycles are vital steps in delaying tumour progression and prolonging survival. Nosocomial infection is the most common complication among hospitalised chemotherapy patients. The age, pre-chemotherapy physical and nutritional status, underlying diseases, clinical staging, pathological types, chemotherapy regimen, chemotherapy cycle, post-chemotherapy white blood cell (WBC) count, invasive procedures and other elements of advanced NSCLC patients can all affect their infection risk and types after chemotherapy [22], with the respiratory system being the primary site of infection; next are the digestive system and urinary system, and Gram-negative bacteria are major pathogens leading to bacterial infection after chemotherapy [23]. It suggests that clinicians should pay attention to evaluating infection risk in advanced NSCLC patients undergoing chemotherapy; during the chemotherapy process, attention should be paid to modulating and supporting its immune function and nutritional status; in the hospitalisation process, attention should be paid to aseptic operation and ward disinfection and sterilisation to reduce colonisation and reproduction of pathogenic bacteria under hospital environmental conditions; for patients with symptoms of infection, broad-spectrum antibacterial drugs should be chosen for empirical therapy to ensure effectiveness of anti-infection treatment.

Herein, we validated the diagnostic value of PCT and NLR in bacterial infections after chemotherapy through comparative analysis. In contrast, NC and CRP had remarkably low diagnostic values, which is basically consistent with the results of previous research [24]. The reason may be that NC and CRP are affected by immune stress elements such as tumours, chemotherapy, etc., and fail to reflect the occurrence of bacterial infections specifically. Though research on biomarkers for bacterial infection has achieved certain progress in recent years, massive infection biomarkers with good application prospects have been screened. Nevertheless, clinical diagnosis of bacterial infection is majorly based on clinical status, imaging manifestations, blood routine, pathogen culture and acute phase protein levels such as PCT and CRP currently; for advanced NSCLC patients receiving chemotherapy, imaging manifestations and WBC count are different from ordinary patients and difficult to apply as a diagnostic basis, and clinical symptoms lack specificity. Furthermore, a pathogenic examination has limitations in terms of a low positive rate and a long period of obtaining results; thus, serum acute phase proteins are crucial diagnostic auxiliary markers. For multiple years, the relationship between PCT and infection has been recognised by the academic community, mainly when severe bacterial infections occur in the body, a marked serum PCT upregulation can be observed; nevertheless, in inflammatory responses and mild infections due to viral infections, autoimmune diseases, organ transplant rejection, etc., serum PCT upregulation is not remarkable; thus, PCT possess high specificity and sensitivity in diagnosis and differentiation of bacterial infections. In relevant research, the diagnostic value of serum PCT for bacterial infections in patients with lung cancer, COPD and other lung diseases has been illustrated. PCT can be applied to guide the application of antibiotics, predicting outcomes and prognosis of disease [25].

In This study, serum PCT demonstrated an AUC of 1.000 in diagnosing bacterial infections among NSCLC patients after chemotherapy, with high sensitivity and specificity. While this finding underscores the strong diagnostic performance of PCT, we acknowledge that an AUC of 1.000 is very rare in clinical research and may reflect potential overfitting or sampling bias. This result could be influenced by factors such as the relatively small sample size, single-centre design, and strict inclusion/exclusion criteria, which may have minimised clinical variability. Further more, bacterial infection diagnoses were based on culture results and imaging findings, which themselves are not perfect gold standards and may contribute to inflated diagnostic estimates. Therefore, we recommend cautious interpretation of this result and emphasise the need for external validation in larger, multicenter cohorts to confirm the generalizability and robustness of PCT’s diagnostic utility in this setting.

NLR is a novel inflammatory marker that has received widespread attention in recent years. NLR is associated with malignancies, sepsis, acute pancreatitis, cardiovascular diseases and other diseases. Since NLR can receive calculations based on blood routine results, it has advantages of convenience, speed, affordability, repeatability, etc., which has good application prospects in communities and grassroots medical institutions. During chemotherapy in advanced NSCLC patients, when changes in traditional blood routine indicators such as WBC cannot indicate infection, the indicative role of systemic inflammatory indicators such as NLR in infection receives highlighting. Scholars have demonstrated that in the diagnosis of bacterial infections after chemotherapy, the diagnostic value of NLR depicts elevation relative to traditional blood routine indicators such as WBC, NC, average neutrophil volume, etc. [26], which is basically consistent with the results of this research. Furthermore, overall survival demonstrated no difference in the infection group, which showed elevation in comparison to those in a non-infection group, suggesting that infection was not an independent risk element for the overall survival of NSCLC patients after chemotherapy. Thus, infection biomarkers are not considered alone in predicting the survival of NSCLC patients.

Thus, infection biomarkers are not considered alone in predicting the survival of NSCLC patients.

Additionally, prior use of antibiotics before biomarker sampling may have influenced the results. Early empirical treatment could suppress infection markers like PCT, CRP, and NLR, leading to an underestimation of their diagnostic value. Since detailed antibiotic timing was not consistently recorded, this remains a potential confounding factor that should be addressed in future studies.

This study has several limitations that may affect the interpretation and generalizability of the results. First, the relatively small sample size from a single institution may limit the applicability of the findings to broader NSCLC populations. Second, the absence of a separate validation cohort restricts the ability to confirm the diagnostic performance of the biomarkers assessed. Third, the timing of biomarker measurements in relation to the exact onset of infection was not consistently defined, which may have influenced the observed marker levels and diagnostic accuracy. Future studies should aim to include larger, multicenter cohorts with well-defined infection timelines and incorporate external validation sets to confirm these findings and enhance their clinical relevance.

## Conclusion

In conclusion, this study compared the diagnostic value of multiple biomarkers. It confirmed the clinical utility of novel indicators such as PCT and NLR in detecting bacterial infections in advanced NSCLC patients undergoing chemotherapy. While NLR demonstrated high sensitivity and PCT showed excellent specificity and prognostic value, their combined use is particularly valuable due to these complementary characteristics. Therefore, when microbiological confirmation is delayed or unavailable, clinicians are encouraged to interpret PCT and NLR together to enhance early diagnostic accuracy and guide timely treatment decisions more effectively.

## Dodatak

### Acknowledgements

The authors would like to express their sincere gratitude to the medical and nursing staff of the Department of Thoracic Surgery at the 4th Centre Hospital of Nankai University and the Department of Pulmonary and Critical Care Medicine at Lishui Second People’s Hospital for their support during data collection and patient care. We also thank all patients and their families for their participation and cooperation in this study.

### Funding

This research received no specific grant from any funding agency in the public, commercial, or not-for-profit sectors.

### Ethics approval and consent to participate

The Medical Ethics Committee of the 4th Centre Hospital of Nankai University approved this study. All participants provided written informed consent prior to inclusion in the study.

### Data availability

The datasets generated and analysed during the current study are available from the corresponding author upon reasonable request.

### Authors’ contributions

Bin Wang contributed to study design, patient recruitment, data collection, and manuscript drafting. Yanbin Wei supervised the study, performed the statistical analysis, and critically revised the manuscript. Both authors read and approved the final manuscript.

### Conflict of interest statement

All the authors declare that they have no conflict of interest in this work.
